# Causal estimation of time-varying treatments in observational studies: a scoping review of methods, applications, and missing data practices

**DOI:** 10.1186/s12874-025-02633-y

**Published:** 2025-08-27

**Authors:** Mercy Rop, Innocent Maposa, Taryn Young, Rhoderick Machekano

**Affiliations:** 1 Division of Epidemiology and Biostatistics, Department of Global Health, Faculty of Medicine and Health Sciences, Stellenbosch University, Cape Town, South Africa; 2 Division of Epidemiology and Biostatistics, School of Public Health, Faculty of Health Sciences, Witwatersrand University, Johannesburg, South Africa

**Keywords:** Time-varying treatments, Time-dependent confounding, Causal inference, Observational studies, Real-world data, Doubly robust, Singly robust, Missing data, Scoping review

## Abstract

**Background:**

Estimating causal effects of time-varying treatments or exposures in observational studies is challenging due to time-dependent confounding and missing data, necessitating advanced statistical approaches for accurate inference. Previous reviews indicate that singly robust methods are prevalent in epidemiological studies despite the availability of more robust alternatives that better handle time-varying confounding. Although common in longitudinal studies, missing data are often inadequately reported and addressed, potentially compromising the validity of estimates. Whether this dependence on less robust methods and inadequate handling of missing data persists in time-varying treatment settings remains unclear. This review aimed to identify current practices, methodological trends, and gaps in the causal estimation of time-varying treatments.

**Methods:**

We conducted a scoping review to map causal methodologies for time-varying treatments in epidemiological studies and identify trends and gaps. To capture the most recent developments, we searched PubMed, Scopus, and Web of Science for articles published between 2023 and 2024. A structured questionnaire was used to extract key methodological aspects, and findings were summarized using descriptive statistics.

**Results:**

Of the 424 articles, 63 met the eligibility criteria, with five added from citations and references, totalling 68 for analysis. Among these, 78% addressed epidemiological questions, 13% included methodological illustrations, and 9% focused solely on methods. Singly robust methods dominated, with inverse probability of treatment weighting (IPTW) being the most common (64.3%), followed by targeted maximum likelihood estimation (TMLE) (14.3%). The emergence of new estimation approaches was also noted. Missing data handling remained inadequate; 33% did not report the extent of missingness, 95.2% lacked assumptions, and sensitivity analysis was performed in only 14.5% of the articles. Multiple imputation (MI) was more prevalent (29%), while complete case analysis (11.3%) was likely underreported, given 33.9% omitted strategy details.

**Conclusion:**

Persistent reliance on singly robust methods, underutilization of doubly robust approaches, and inadequate missing data handling highlight ongoing gaps in evaluating time-varying treatments. While newer estimation approaches are emerging, their adoption remains limited. These trends, alongside the growing complexity of real-world data and the demand for evidence-driven care, call for greater methodological rigor, wider adoption of robust approaches, and enhanced reporting transparency.

**Supplementary Information:**

The online version contains supplementary material available at 10.1186/s12874-025-02633-y.

## Introduction

Time-varying treatment plans are increasingly utilized in clinical settings to deliver personalized care to patients with chronic and progressive medical conditions [1]. These plans involve continually monitoring and evaluating a patient’s evolving disease state and treatment responses, enabling dynamic adjustments to treatment decisions for optimized care. Similarly, many epidemiological exposures of interest are often time-varying and may influence clinically relevant outcomes differently over time [2].

**Fig. 1:**
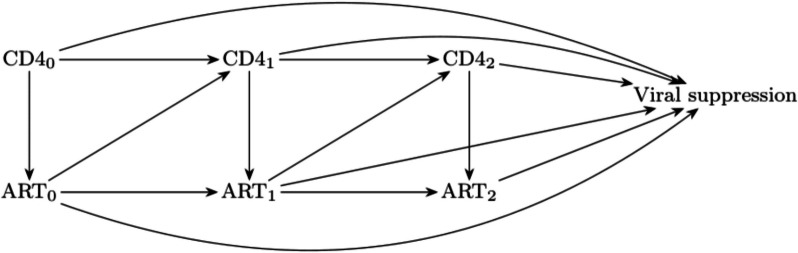
Treatment-affected time-varying confounding over three time points

When treatments or exposures change over time, treatment-affected time-varying confounding can occur because of evolving covariates and treatment history. This phenomenon occurs when earlier treatments influence the patient’s disease state, which in turn influences subsequent treatments and outcomes of interest [3, 4]. For instance, before the implementation of the universal test-and-treat policy in HIV management in 2016 [5], antiretroviral therapy (ART) was initiated only once CD4 counts dropped below a predefined threshold. However, throughout the follow-up period, earlier ART influenced CD4 counts, as successful treatment led to immunological recovery. This interplay creates a feedback loop in which CD4 counts dictate treatment decisions, treatments alter CD4 counts, and both factors ultimately affect clinical outcomes. This dynamic interplay is illustrated by a directed acyclic graph (Fig. 1).

Standard regression-based adjustment (RBA) analyses often fail to address these feedback loops adequately because they condition confounders affected by prior treatment, which can introduce bias and result in misleading conclusions [3]. Additionally, missing data, ubiquitous in longitudinal observational studies due to attrition, dropouts, non-adherence, and data collection challenges, further complicate accurate causal estimation in time-varying treatment settings [5, 6].

Unlike randomized controlled trials (RCTs), treatment assignments in observational settings are non-random and usually influenced by patient characteristics and physician decisions. Consequently, the probability of receiving a specific treatment or treatment sequence is not known a priori and must be estimated. Causal inference in these settings is commonly approached within the potential outcomes framework [7], which posits the existence of multiple potential outcomes for each treatment option (level). Within this framework, specialized confounding adjustment methods are used, and key identifiability assumptions—such as non-interference, consistency, positivity, and exchangeability—must be made and satisfied for the estimated effects to be interpreted causally [2, 8].

Over the past three decades, several statistical methods have been developed to adjust for treatment-affected time-varying confounding and estimate the average treatment effects (ATE). These methods include the g-formula, g-estimation with Structural Nested Mean Models (SNMM), and Inverse Probability of Treatment Weighting (IPTW) of Marginal Structural Models (MSMs), collectively developed by Robins et al. [4] and referred to as the g-methods, as well as doubly robust methods, such as Augmented Inverse Probability of Treatment Weighting (AIPTW) [9] and Targeted Maximum Likelihood Estimators (TMLE) [10]. A comprehensive review of these methods is provided elsewhere [8, 10–13].

Briefly, the g-methods rely on correctly estimating either the outcome or treatment model for unbiased estimation of ATE. However, incorrectly specified models can lead to biased estimates and misleading conclusions [9]. Doubly robust (DR) methods aim to address this by requiring the specification of both the treatment and outcome models, and unbiased estimates can still be obtained when either model is misspecified but not both. These DR methods are also efficient when both models are consistently estimated [9].

While IPTW methods used in Marginal Structural Models (MSMs) effectively address time-varying confounding, standard implementations may not explicitly model interactions between treatments and time-varying covariates without additional specification in the MSM model [4]. Nonetheless, their resemblance to standard statistical procedures makes them intuitive and widely used in applied epidemiological research [10, 14]. Moreover, accurate specification of the treatment model is critical to avoid bias, yet traditional g-methods often rely solely on domain knowledge for this specification, which may not guarantee accurate estimation in high-dimensional observational settings with several covariates varying over time [8, 14]. To address these limitations, efforts to enhance g-methods by incorporating machine learning techniques are ongoing [15–18]. These advancements aim to make the methods more data-adaptive, flexible, and accurate, enabling better capture of complex relationships among variables.

Although AIPTW enhances the IPTW methodology by incorporating an augmentation term that corrects the estimator when the treatment model is misspecified, the estimator can perform poorly in the presence of extreme propensity scores [19]. Additionally, challenges related to non-unique solutions in its estimating equations and not restricting the ATE estimate to the parameter space make it difficult to implement and interpret, limiting its widespread use [20].

In contrast, some doubly robust methods, such as TMLE, protect against model misspecification and include a targeting step that reduces the bias-variance trade-off, enabling more accurate estimates. Additionally, TMLE can seamlessly integrate Super Learner, an ensemble machine learning algorithm that combines multiple learners to identify and leverage the best-fitting model to improve efficiency [21].

Although TMLE can be implemented without Super Learner, it is generally considered best practice to use it to enhance performance [22]. In high-dimensional data with numerous time-varying covariates, this combination can uncover complex patterns and relationships among variables in the data [19], including non-linear, interaction, and higher-order relationships, thus improving model estimation. Previous comparative assessments have also demonstrated the superiority of TMLE methods over single robust alternatives in terms of efficiency and ability to simultaneously reduce bias and variance [23].

Since its introduction in 2006 [10], the TMLE methodology has undergone tremendous growth and advancement [24], broadening its scope and application to diverse settings, including longitudinal observational studies [19, 23, 24], time-to-event outcomes [25], clustered data [22] and, more recently, to dynamic treatment regimens or adaptive strategies [26].

Similarly, causal machine learning (CML) methods are gaining prominence for estimating treatment effect heterogeneity (HTE) [24, 27–29]. By using machine learning algorithms to uncover variations in treatment response, CML approaches such as causal forests provide insights into treatment efficacy across patient subgroups, enabling more refined personalized strategies and policies [30, 31].

Additionally, the increasing availability of observational data from large electronic databases, advances in causal inference and computing methodologies, and the growing need for evidence-based healthcare have driven efforts to develop methods that combine findings from RCTs and observational studies [29, 32, 33]. This integration seeks to leverage the strengths of both designs to generate high-quality evidence for better health care.

### Methodological trends and challenges in causal inference in epidemiological studies

In a systematic review conducted in 2019 [27], it was found that many applied researchers continue to rely on older, simpler, and less robust methods for evaluating time-varying treatments, despite the availability of more sophisticated alternatives, such as TMLE and AIPTW. A subsequent review in 2021 [34] pointed out a similar reluctance to embrace newer methods and the continued dominance of familiar traditional approaches. However, by 2022 [24], a systematic review noted a gradual increase in the use of TMLE in epidemiological studies, with further uptake anticipated as accessible tutorials and software packages become more widely available beyond the methodological community.

Furthermore, the 2021 review [34] identified gaps in the methodological quality of published studies that evaluated optimal time-varying treatment plans in observational studies. One prevalent issue was the inadequate handling of missing data, with approximately 51% of the studies failing to transparently report the strategies used to address missing data, contrary to reporting guidelines for observational studies [34, 35]. Similarly, Carroll et al. [36] explored how missing data are handled in time-to-event analyses for observational oncology studies. They found that simple strategies such as complete case analysis (CCA) were still the most used (53%), while more sophisticated methods such as multiple imputation (MI) were employed in only 22% of the studies. Missing data assumptions were rarely reported (14%), and sensitivity analyses were conducted in just 7% of cases, limiting the reliability of the study findings.

Similarly, a more recent systematic review [37] assessed gaps in the reporting and use of MI when addressing causal questions in observational studies with missing data across multiple variables. They noted that most studies did not report missing data, associated assumptions, or analysis decisions with sufficient clarity; in cases where missing data assumptions were provided, proper justifications were lacking. For instance, the common justification for MI implementation was the Missing at Random (MAR) assumption, often without thorough assessments or sufficient rationale to support the claim [37].

This underappreciation and underreporting of missing data have implications for the validity of conclusions, as selection bias and information loss can be introduced, obscuring the true effects of treatments on clinically relevant outcomes. Therefore, appropriate handling of missing data is essential for unbiased and accurate causal estimates.

### Strategies for handling missing data

Various strategies are available for analyzing incomplete data, each suited to different missing data mechanisms or assumptions regarding the reasons for the missing data [38–41]. Missing data are commonly classified according to Little and Rubin’s framework [42] as Missing Completely at Random (MCAR), Missing at Random (MAR), or Missing Not at Random (MNAR).

Under the assumption of MCAR, CCA yields unbiased estimates because the subsample remains representative of the study population [43]. However, when MCAR does not hold, CCA may introduce selection bias, as complete cases may differ systematically from those with missing data, leading to non-representativeness.

Multiple imputations are generally recommended when the MAR assumption is plausible [36, 41]. It generates multiple complete datasets by imputing missing values using a predictive probability distribution derived from observed data. These datasets are then analyzed separately, and the results are pooled using Rubin’s rule, which ensures that both between- and within-imputation variations are accounted for in estimating the standard errors.

Multiple imputation is increasingly used in epidemiological studies [37] and is often considered the'gold standard'because it mitigates information loss by using all available data and accounts for the uncertainty around missing data, yielding more precise estimates [39, 42, 43]. However, consistent MI estimation depends on the correct specification of imputation models [44]. Essentially, the models need to have all the variables that are intended to be part of the analysis incorporated in the model in a manner that depicts the actual relationship between the variables; failure to do so may lead to incompatibility between the imputation model and the analysis model, resulting in biased estimates [45, 46]. Fortunately, flexible data-adaptive MI approaches that combine machine learning algorithms are currently available to improve the chance that imputation models are appropriately specified [47–49].

Single imputation approaches, such as using the mean or median of observed data or carrying forward the last observed value (LOCF), are prevalent in epidemiological studies. These methods impute a single value for each missing observation, assuming that the imputed value accurately represents the true missing values. Hence, failing to account for the uncertainty in missing data and underestimating standard errors can lead to biased estimates and incorrect inferences [43]. Consequently, these methods are widely discouraged as primary strategies for handling missing data [50–53]. However, LOCF may be considered reasonable under certain strong justifications that the last observed value reflects the most recent information guiding treatment decisions, particularly when the condition is expected to remain stable or the treatment effect is unlikely to change substantially [54].

Missing data challenges can also be addressed using inverse probability weighting (IPW) or its extension for censoring in time-to-event analyses, known as the inverse probability of censoring weighting (IPCW). These techniques create a pseudo-population in which the observed data accurately reflect the entire target population, particularly when all relevant population characteristics or conditioning variables are included in the observed data. They achieve this by assigning greater weight to individuals who are underrepresented because of missing data or censoring. Each individual receives a weight that is the inverse of the estimated probability that their data are observed given their covariates and history. This method reduces bias under the MAR assumption by ensuring that the analysis properly considers the differential probabilities of observation [6, 49].

Additionally, within the TMLE framework, there is growing literature on handling missing covariate data by incorporating a missing data indicator (1 if observed, 0 if missing) into the nuisance parameter models [55–57]. In this context, the target parameter is redefined under a hypothetical intervention where everyone is observed, allowing estimation under complete data scenarios. Combined with Super Learner, this approach enables a flexible, data-adaptive approach for handling missing data. This extension is explicitly implemented in Longitudinal TMLE via the ltmle package in R [58].

Finally, in situations where missing data are assumed to be MNAR, more advanced approaches, such as selection models [59], pattern-mixture models [60] and Bayesian approaches [43, 51], are recommended.

There is no universally best method for handling missing data in all situations; instead, the choice of approach should be guided by the specific nature and context of the missing data [51]. After handling missing data, it is important to conduct sensitivity analyses to assess the robustness of the findings to potential deviations from the assumed missing data mechanism in the primary analysis, mainly because the assumptions made cannot be verified from the observed data [6, 43, 53, 54]. This ensures that research findings provide a more transparent picture of the uncertainty surrounding the results.

The effective use of robust causal methods, in conjunction with appropriate handling of missing data, can significantly improve the accuracy of insights into the personalized management of chronic and progressive diseases. However, the extent to which applied researchers have embraced these advanced methods and whether appropriate reporting and optimal handling of missing data have been adequately incorporated into evaluating treatment effects of time-varying treatments in epidemiological studies remains unclear.

## Objectives of the review

This scoping review builds on prior reviews [14, 22, 61, 62], evaluating the utilization of causal methods and progress in adopting advanced causal techniques in epidemiological studies. It focuses specifically on their application in time-varying settings and identifies the remaining gaps in methodological practice, particularly concerning the reporting and handling of missing data in observational studies. The specific objectives were to:

- Identify and describe methodologies used to estimate the average treatment effects of time-varying treatments and exposures.
- Assess the extent to which the amount of missing data was reported.
- Evaluate the extent to which missing data assumptions were explored and reported.
- Identify strategies employed to handle missing data.
- Determine whether sensitivity analyses were conducted to assess the robustness of missing data assumptions.

## Methods

This scoping review followed the Preferred Reporting Items for Systematic Reviews and Meta-Analyses extension for Scoping Reviews (PRISMA-ScR) checklist [63] and adhered to relevant guidelines [54, 64]. The literature reviewed in this study included articles published between January 1, 2023, and December 31, 2024. The inclusion of more recent literature allows this review to update and extend prior reviews [14, 22, 61], capturing the most current trends, gaps, and practices in the epidemiological field.

### Search strategy

We systematically searched PubMed, Scopus, and Web of Science databases on January 5, 2025, to identify primary research articles published within the defined period. The search strategy was developed in collaboration with a skilled librarian, who assisted in constructing the search terms. The search terms consisted of three main components: time-varying treatments, adjustment methods, and observational studies. The primary search terms were built as an ordered combination of the three key components, with synonyms and related phrases to capture all relevant variations (Table 1).

**Table 1:** Search terms

Key terms	Synonyms
Time-varying treatments	Adaptive interventions, dynamic treatment strategies, time-dependent confounding, treatment policy/treatment policies
Causal methods	Adjustment methods, TMLE, IPTW, AIPTW, SNMM, G-estimation, g-formula, g-computation formula, Marginal structural models (MSMs), causal machine learning, causal AI, causal deep learning
Observational studies	Non-randomized, non-experimental, real-world data, electronic medical records, electronic health records

The search terms were tailored for each of the three databases. The full PubMed search strategy is shown in Table 2, with strategies for the other databases provided in Additional file 1.

**Table 2:** PubMed search terms

Search	Search combination used
#1	“Dynamic treatment regimens” OR “Dynamic treatment regime*” OR “Dynamic treatment strategies” OR “Adaptive intervention*” OR “adaptive strateg*” OR “adaptive treatment*” OR “time-varying” OR “time-dependent” OR “treatment policy” OR “treatment policies” OR “Sequential treatment decisions”
#2	“Causal” OR “Causal inference” OR “Targeted Maximum Likelihood Estimation” OR “TMLE” OR “targeted maximum likelihood estimation”) OR “targeted minimum loss based estimation” OR “targeted minimum loss-based estimation” OR “targeted machine learning” OR “targeted learning” OR “targeted machine-learning” OR “Inverse Probability of treatment weighting” OR “IPTW” OR “Augmented Inverse Probability of Treatment Weighting” OR “AIPTW” OR “Structural Nested Mean Models” OR “SNMM” OR “G-Estimation” OR “g-formula” OR “g-computation formula” OR “Marginal structural model” OR “Causal AI” OR"causal ML"OR"Causal deep learning"OR"Bayesian causal"
#3	“Observational” OR “Non-randomized” OR “non-randomised” OR “real-world*” OR “Electronic medical records” OR “EMR” OR “Electronic health records” OR “EHR”
#4	#1 AND #2 AND #3

### Eligibility criteria

We included articles proposing or applying statistical methods for time-varying treatments in observational settings, including peer-reviewed articles and conference proceedings published between 2023 and 2024, as well as grey literature such as technical reports and preprints not formally published in peer-reviewed journals. The inclusion criteria required articles to address causal questions related to time-varying treatments in observational settings, or they propose, illustrate, or implement methods for adjusting for time-varying confounding. Additionally, only publications in the English language, with both abstract and full-text available, were included. Articles were excluded if they were review papers, opinion articles or commentaries, study protocols, simulations, or comparative studies.

### Selection of relevant articles

The search output was imported into the Rayyan software [65] to organize and manage the literature and facilitate team collaboration while selecting relevant articles. The initial screening was conducted based on titles and abstracts, followed by a detailed assessment of full articles for eligibility. Additional articles were selected based on the citations and references of the included studies to ensure thorough coverage. Two authors independently reviewed the articles.

Data on the fields listed in Table 2 were extracted using a standardized form (in Microsoft Excel) for each article. Data extraction was performed by MR and confirmed by RM (Table 3).

**Table 3:** Data extraction

Item	Item definition
Complete reference	Title, Journal name, year of publication
Paper focus	The paper's focus: methodology or clinical questions
The clinical focus	The medical condition of the paper, e.g., HIV, cancer
Causal question	Whether the research question is causal or associational
Statistical adjustment methods used	The methods used to adjust for confounding, e.g., IPTW
The extent of missingness described	Amount of missing data reported either in text or in a table/figure
Missing data mechanism	Missing data assumptions explored and reported
Handling of missing data	Strategies employed to address missing data and their justifications
Sensitivity analysis	Whether sensitivity analyses were done to assess the robustness of missing data assumptions. If yes, which methods were used

### Data analysis and reporting

The extracted data were analyzed using descriptive statistics (frequencies and percentages) in Stata 18 [66], narratively synthesized and reported following established guidelines [63].

## Results

**Fig. 2:**
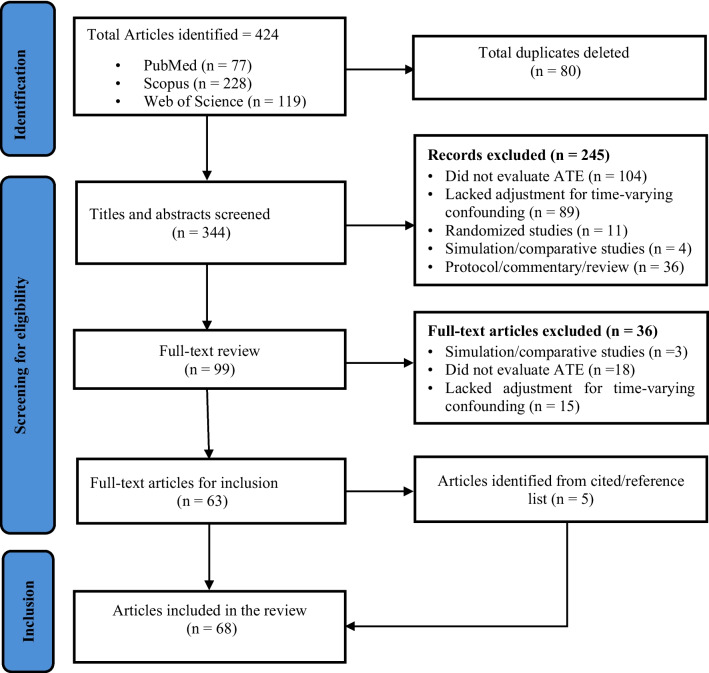
Flowchart outlining article identification and inclusion in the scoping review

The initial search yielded 424 articles. After removing duplicates, 344 studies were screened by titles and abstracts, and 99 advanced to full-text reviews. Of these, 63 studies met the inclusion criteria [67–129], with five additional studies [130–134] identified through citation tracking and reference lists. In total, 68 articles were included (Fig. 2), with 36 (53%) published in 2023 and 32 (42%) in 2024.

Most studies (53, 78%) focused on answering clinical and epidemiological questions, while nine (13%) focused primarily on methodology alongside illustrative analyses using real-world data, and six (9%) focused on methodological concepts only (Table 4).

**Table 4:** Characteristics of reviewed papers

Characteristics	Total (*n* = 68)
Year of publication	
2023	36 (52.9%)
2024	32 (47.1%)
Focus of the paper	
Answer clinical questions	53 (77.9%)
Methodology + illustrative analysis	9 (13.2%)
Methodology only	6 (8.8%)
Clinical focus area	
COVID-19	6 (9.7%)
Dentition	5 (8.1%)
Mental health disorders	6 (9.7%)
Mortality	5 (8.1%)
Cancer	4 (6.5%)
HIV	4 (6.5%)
Diabetes	3 (4.8%)
Kidney issues	4 (6.5%)
Other	25 (40.3%)
Disease category	
Chronic & progressive conditions	31 (54.4%)
Other	26 (45.6%)
Methods used	
Propensity score methods	39 (59.1%)
TMLE	6 (9.1%)
G-formula	6 (9.1%)
G-estimation	4 (6.1%)
Bayesian approaches	3 (4.5%)
Instrumental variables	2 (3.0%)
ML	3 (4.5%)
Other	3 (4.5%)
Causal method category (Application papers)	
Singly robust	56 (90.3%)
Doubly robust	6 (9.7%)
Extent of missingness reported	
No	21 (33.9%)
Yes, in a table/figure	19 (30.6%)
Yes, in text	22 (35.5%)
Missing data assumptions reported	
No	59 (95.2%)
No, but implied by text as MAR	1 (1.6%)
Yes, MAR	1 (1.6%)
Yes, MNAR	1 (1.6%)
Missing data strategy	
MI	18 (29.0%)
CCA	7 (11.3%)
SI	10 (16.1%)
Multiple approaches	6 (9.7%)
Not indicated	21 (33.9%)
Sensitivity analysis done	
No	53 (85.5%)
Yes	9 (14.5%)

The estimation of the causal effects of dynamic treatments and time-varying exposures spanned various health conditions. Key focuses included COVID-19 (6, 9%), mental health disorders (6, 9%), dentition (5, 7.4%), and cancer and HIV (4 studies each, 6%).

Mortality (*n* = 5, 7.4%) was a frequent outcome of interest. Although COVID-19 accounted for the largest proportion, studies addressing chronic and progressive conditions collectively comprised a significant share (31 studies, 54%), underscoring the growing importance of dynamic treatment regimens and time-varying exposures in epidemiological research.

### Causal methods

Among the methodological papers, the emphasis was on demonstrating the theory and application of emerging concepts, such as machine learning [56, 58, 88], proximal approaches to address unmeasured confounding [71], and the use of high-dimensional propensity scores [81, 123], an extension of the traditional propensity score that enables an automated, data-driven approach to covariate selection. This trend highlights the increasing role of data-driven methodologies in causal estimations.

In the application and methodology illustrative papers, propensity score methods (*n* = 39, 59%), particularly IPTW, were the most frequently used, appearing in 27 studies (44%). TMLE was used in six studies (9.7%), followed by the g-formula in five studies (8.1%), and G-estimation in three studies (5%). Bayesian approaches, along with instrumental variables, joint models, and machine learning techniques, were the least common. Regarding robustness, doubly robust methods were employed in only 6 studies (14.3%), whereas the majority (56 studies, 90.3%) relied on singly robust methods.

### Missing data reporting and handling

In addition to the range of causal estimation methods, we observed substantial variability in reporting and handling missing data. Among the 62 application papers, 21 (33.9%) did not report the extent of missing data, while 19 (30.6%) provided details in tables and figures, and 22 (35.5%) merely mentioned missingness briefly in the text without offering precise details, sometimes using vague statements such as “ < 5% missingness in all variables,” and “there was small missingness in the covariates.” While these statements provide some indication of the extent of missing data, they lack specificity and leave questions regarding the underlying assumptions and potential bias unaddressed.

Regarding missing data assumptions, most studies (59, 95.2%) did not explicitly state any assumptions regarding missingness in the data. Only two studies (3.2%) assumed that missing data were MAR, and a single study (1.6%) clearly stated that the missing data were MNAR. About one-third of the studies did not indicate a missing data strategy utilized, which may suggest the use of CCA, as this is the default in most statistical packages. Among the reported strategies, MI was the most used, applied in 29% [17] of the studies.

Single Imputation (SI) approaches, including LOCF and mean or median imputation, were employed in ten studies (16.1%). LOCF was also frequently used in studies that employed multiple strategies (six studies, 9.7%). However, only 9 studies (14.5%) conducted sensitivity analyses to assess the robustness of the results to missing data assumptions, while the majority (53 studies, 85.5%) did not perform such analyses.

## Discussion

In this scoping review, we provide an up-to-date overview of the methodological practices in estimating the average treatment effects of dynamic treatment regimens and time-varying exposures in observational epidemiological studies, focusing on causal methodologies and handling missing data. We observed significant variations in approaches used for causal estimation and the reporting and management of missing data across the 68 included studies, which covered a wide range of health conditions. We also noted an increase in the use of new analytical techniques.

Previous reviews [14, 61] had indicated a primary focus of time-varying regimens on HIV and chronic conditions such as cancer. While the current review highlights a continued emphasis on chronic diseases (31 studies, 54%), there was a noticeable shift towards acute conditions, particularly COVID-19, reflecting the field’s responsiveness to emerging public health crises.

Our findings also indicate that singly robust methods, particularly propensity score-based approaches such as IPTW, continue to dominate the evaluation of time-varying treatments, while doubly robust methods remain underutilized. This reflects a continued reliance on traditional singly robust approaches in current practice, consistent with previous reviews [14, 61], which also reported the predominance of IPTW methods, likely owing to their ease of implementation and interpretability [135], suggesting a reluctance to adopt newer methods. However, despite expectations of increased TMLE adoption [136], the current review noted a slow uptake. The limited use of doubly robust methods in real-life applications presents an opportunity to improve causal inference in complex data settings, such as those considered in this review.

Shifting to robust methods is likely perceived as challenging because of the complexity of their modeling approaches and computational demands. In particular, both the Super Learner and multiple imputation (MI) can be resource-intensive, especially when applied to large datasets. While specialized software options are steadily improving within open-source platforms such as R, comparable support remains limited in other widely used statistical packages such as Stata and SAS.

For new users, these tools can be intimidating because of their steep learning curves and limited troubleshooting support. Although efforts have been made to enhance accessibility through lay-language tutorials for applied researchers [19, 137] and the availability of freely available implementation packages in R, ongoing efforts are needed to develop and maintain user-friendly tools and documentation to support broader adoption in epidemiological research. For instance, the tlverse software suite for TMLE (GitHub: https://github.com/tlverse/tlverse), which has not been updated in the last four years, provides a platform that can be further enhanced for improved accessibility by non-specialist users. Additionally, the rapid pace of development in the causal inference landscape reinforces the need for continued collaboration between methodologists and applied scientists to bridge the gap between theory and practice [24], as methodological advancements alone, without practical implementation, cannot drive meaningful progress.

Historically, non-Bayesian approaches have dominated the theory and practice of causal estimation in epidemiological studies [72]. This trend will likely persist in the current practice due to theoretical, computational, and programming requirements that remain inaccessible to domain experts. Along with the growing interest in integrating machine learning techniques with frequentist causal methods [123, 126], similar efforts are gaining traction within Bayesian causal inference frameworks [72, 138].

The potential for unmeasured time-varying confounding is also a significant concern in time-varying observational settings, as it can bias estimates [139]. As a result, methodologies that use proximal approaches are being developed [71]. A related trend is the increasing use of high-dimensional propensity scores for covariate selection [93]. These newer innovative approaches are expected to increasingly shape the causal inference field, particularly with the increasing complexity of real-world clinical data, the rising demand for evidence-driven care, and the advancements in statistical computing.

Missing data remains a persistent challenge in longitudinal observational studies; however, it was not adequately reported in many studies. Notably, slightly more than one-third failed to disclose the extent of missing data for each variable, while the other third provided only limited details. This is consistent with the findings of a previous review [34], which noted that up to half of the studies provided no information regarding the amount of missingness in the data. The main issue arising from missing data is selection bias, which becomes more pronounced as the proportion of missing data increases. As a result, this lack of transparency regarding the extent of missing data limits the ability to assess the validity of the findings and complicates the comparison of results across studies. Despite the guidelines for reporting observational studies [62, 140] emphasizing clear and explicit reporting of missingness, adherence was mostly inconsistent.

Our findings also reveal that assumptions underlying missing data are rarely explored and stated. This was similar to previous reviews [62, 140], which noted infrequent reporting of missing data assumptions, with approximately 70–86% failing to provide a statement on assumptions made about the underlying missingness in the data. Assumptions regarding the mechanism of missing data can significantly influence the estimates obtained from statistical analyses because different approaches are appropriate for different assumptions [41]. Failure to consider them in the analysis may lead to incorrect inferences or underestimation of the uncertainty surrounding missing data.

This scoping review also highlights a varied approach to addressing missing data. The most common strategy was MI, which is an improvement from previous reviews [61, 62] where CCA was predominant. Although this review shows that MI is becoming more widely used, CCA may still be the preferred method in practice, particularly in studies that do not explicitly report their missing data strategies. Despite its simplicity and widespread use, CCA can potentially introduce selection bias when the missing data are not MCAR.

Interestingly, some studies employed multiple missing data strategies, often combining LOCF, a common bedside practice in clinical settings, with other methods. Although combining multiple approaches may reflect a more deliberate effort to address missing data, it can also complicate the interpretation of results because it introduces variability and inconsistency in how missing data are handled across the dataset.

Another notable concern is the limited use of sensitivity analyses to assess the robustness of missing data strategies. This raises questions regarding the validity and reliability of the conclusions drawn from such studies, as the potential impact of missing data may not have been assessed thoroughly.

The underappreciation and under-reporting of the extent of missing data, assumptions made, inadequate handling, and lack of sensitivity analyses point to a broader issue of insufficient reporting in observational research, a finding similar to that of a recent review by Mainzer et al. [37], where they called for increased concerted efforts to improve transparency in reporting missing data practices. Given the challenges of time-varying confounding, the prevalence of missing data in multiple covariates (potential confounders) in observational studies, and their potential impacts on estimates, more detailed and transparent reporting of both the methods used and the assumptions made is essential for enhancing the reproducibility and robustness of causal estimates.

The strength of this study lies in its thorough evaluation of causal methodologies across a large body of literature spanning three databases, offering a clear picture of current trends and practices in applied epidemiology over the past two years. Although potentially relevant articles may have been missed, we took extensive measures to identify all relevant studies by examining cited and citing articles, which were few due to the recent review period (2023–2024).

Overall, although time-dependent confounding and missing data are distinct methodological challenges, they often intersect in observational studies. Our findings highlight persistent gaps in the reporting and handling of missing data in these studies. These gaps are particularly concerning, given the frequent reliance on singly robust methods, which are more prone to bias due to their dependence on a single model. Combined with inadequate reporting and handling of missing data, the impact of this bias can be significantly amplified, potentially leading to misleading conclusions about treatment effects. With the rise in personalized medicine and the increasing volume and complexity of real-world healthcare data, the need for rigorous methodologies has never been greater.

## Conclusion

Time-varying treatments and exposures of epidemiological interest are increasingly common, making proper adjustments for time-varying confounding and handling of missing data essential for accurate estimation. This review highlights the widespread reliance on singly robust methods in causal inference for time-varying treatment settings, despite the availability of more robust alternatives, alongside significant gaps in the reporting and management of missing data. Addressing these dual challenges by implementing appropriate, more modern, and rigorous methodologies and improving transparency, as recommended in guidelines for observational studies, will move causal inference for time-varying treatments in observational settings to greater validity and reliability.

Moreover, the growing integration of machine learning (ML) and artificial intelligence (AI) techniques offers innovative solutions for causal inference challenges, particularly in complex, high-dimensional settings and in estimating treatment effect heterogeneity. These techniques provide greater modeling flexibility and reveal intricate patterns and relationships that traditional methods may fail to detect. However, their application in time-varying settings is still evolving.

Finally, with the growing interest in developing methods that integrate findings from RCTs and observational studies, as well as those that account for residual confounding, it would be valuable to explore their implementation and relative performance in time-varying settings in the future.

## Supplementary Information

Supplementary Material 1.

## Data Availability

The datasets used and analyzed during the current study are available from the corresponding author upon reasonable request.

## Abbreviations

ATE: Average treatment effects; ART: Anti-retroviral therapy; CCA: Complete case analysis
CML: Causal Machine Learning; DR: Doubly robust
HTE: Heterogeneous Treatment Effects; SNMM: Structural nested mean model
IPTW: Inverse Probability of Treatment Weighting
IPW: Inverse Probability of Weighting; IPCW: Inverse Probability of Censoring Weighting
AIPTW: Augmented Inverse Probability of Treatment Weighting
TMLE: Targeted Maximum Likelihood Estimation/minimum-loss based estimation
MCAR: Missing data can be classified as Missing Completely at Random
MAR: Missing at Random; MNAR: Missing Not at Random
MI: Multiple Imputation; LOCF: Last observation carried forward
HIV: Human Immunodeficiency Virus; RBA: Regression-based adjustment
RCTs: Randomized Controlled Trials; SAS: Statistical Analysis System
