# Supplementary material for: Causal estimation of time-varying treatments in observational studies: a scoping review of methods, applications, and missing data practices
Source: BMC Med Res Methodol. 2025 Aug 27;25:202. doi: 10.1186/s12874-025-02633-y (PMC12382241; doi:10.1186/s12874-025-02633-y)
Supplement: Supplementary file 1 — Supplementary Material 1. [file 12874_2025_2633_MOESM1_ESM.docx]

**Additional file 1.**

**Search terms and strategy used for Scopus and Web of Science**

1. **Scopus**

| **Search** | **Search combination used** |
| --- | --- |
| #1 | “Dynamic treatment strategies” OR “Adaptive interventions” OR “adaptive strategies” OR “adaptive treatments” OR time-varying treatments OR time-dependent confounding OR “treatment policy” OR “treatment policies” **AND** “Causal” OR “Targeted Maximum Likelihood Estimation” OR “targeted maximum likelihood estimation” OR “targeted minimum loss based estimation” OR “targeted minimum loss-based estimation” OR “targeted machine learning” OR “targeted learning” OR “targeted machine-learning” OR “Inverse Probability of treatment weighting” OR “Augmented Inverse Probability of Treatment Weighting” OR “Structural Nested Mean Models” OR “G-Estimation” OR “g-formula” OR “g-computation formula” OR “Marginal structural model” OR “Causal AI” OR "causal deep learning" OR “causal ML" OR "Bayesian Causal" **AND** “Observational studies” OR “Non-randomized studies” OR “real-world data” OR “real-world evidence” OR “Electronic medical records” OR “Electronic health records” |

1. **Web of Science**

| **Search** | **Search combination used** |
| --- | --- |
| #1 | “Dynamic treatment regimens” OR “Dynamic treatment regimes” OR “Dynamic treatment strategies” OR “Adaptive intervention*” OR “adaptive strategies” OR “adaptive treatment*” OR “time-varying” OR “time-dependent” OR “treatment policy” OR “treatment policies” OR “Sequential treatment decision*” |
| #2 | “Causal” OR “Targeted Maximum Likelihood Estimation” OR “TMLE” OR “targeted maximum likelihood estimation”) OR “targeted minimum loss based estimation” OR “targeted minimum loss-based estimation” OR “targeted machine learning” OR “targeted learning” OR “targeted machine-learning” OR “Inverse Probability of treatment weighting” OR “IPTW” OR “Augmented Inverse Probability of Treatment Weighting” OR “AIPTW” OR “Structural Nested Mean Models” OR “SNMM” OR “G-Estimation” OR “g-formula” OR “g-computation formula” OR “Marginal structural model” OR “Causal AI” OR "causal ML" OR "causal deep Learning" OR "Bayesian Causal" |
| #3 | “Observational” OR “Non-randomized” OR “non-randomised” OR “real-world data” OR “real-world evidence” OR “Electronic medical records” OR “EMR” OR “Electronic health records” OR “EHR” |
| #4 | #1 AND #2 AND #3 |
